# Epidemiology of physical activity and sedentary behavior levels among patients entering treatment for substance use disorder in the United States: a descriptive study

**DOI:** 10.3389/fpsyt.2024.1348047

**Published:** 2024-04-24

**Authors:** Sydney L. Churchill, Jeni E. Lansing, Angelique G. Brellenthin, Jacob D. Meyer

**Affiliations:** Department of Kinesiology, Iowa State University, Ames, IA, United States

**Keywords:** sedentary, physical activity, behavior, substance use disorder, treatment

## Abstract

**Introduction:**

Little is known about physical activity behaviors among people with SUD. This study aimed to (a) describe self-reported moderate-to-vigorous physical activity (MVPA) and sedentary (SED) behaviors of adults with SUD initiating treatment (b), determine the potential contributions of drug of choice (DOC) on these behaviors, and (c) determine the potential contributions of level of care and demographic variables on these behaviors.

**Methods:**

Secondary data that was collected via surveys including demographic information, psychological health, drug of choice, MVPA (categorized as inactive, insufficiently active, meets guidelines, exceeds guidelines) and SED (<4 h/day, 4-<6 h/day, 6-8 h/day, >8 h/day) were analyzed from 1,293 patients in inpatient/outpatient treatment facilities across the United States.

**Results:**

On average, over half (51%) of patients entering treatment reported not meeting guidelines, but sitting time was generally low (median= 360 min/day). MVPA levels differed based on level of care (p<0.001) with 48% of patients in detox facilities reporting inactivity compared to 37% in residential and 29% in outpatient programs. MVPA and SED levels differed by sex with women less likely to report sitting <4 h/day (27.9% vs. 38.2%, p<0.001) and more likely to report sitting for >8 h/day (31.5% vs. 21.8%, p<0.001) compared to men. SED differed by race (p=0.01), with 54% of Black patients reporting <4 h/day compared to 33% of White patients.

**Discussion:**

Understanding activity behavior patterns among individuals entering SUD treatment provides opportunities for identifying the extent of lifestyle behavior needs and opportunities to develop personalized treatment strategies.

## 1 Introduction

Substance use disorder (SUD) is a mental illness that results in compulsive use of a substance (e.g., alcohol or drugs) which can lead to harmful psychological and physical changes (e.g., cognitive impairment, harm to vital organs; 1). According to the 2020 National Survey on Drug Use and Health report, 40.3 million people aged 12 or older in the United States had a SUD in the past year, with 28.3 million people having an alcohol use disorder, 18.4 million people having a drug use disorder, and 6.5 million people having both (2). An estimated 1.4% (4.0 million people) received substance use treatment in 2020, 17.2% (362,000 people) received medication-assisted treatment for alcohol use, and 30.5% (798,000 people) received medication-assisted treatment for opioid use (2). Thus, SUD is a major public health concern.

Physical activity may be beneficial in alleviating psychological and physical symptoms associated with SUD, however there is limited data on current activity behaviors of those with SUD entering treatment. Understanding moderate-to-vigorous physical activity (MVPA) or sedentary (SED) behavior and SUD relationships is important as increasing physical activity could be a promising adjunctive treatment option for reducing substance use (3). However, many of the observational studies that have examined the relationship between activity behaviors and substance use have been conducted with adolescents or young adults with inconsistent activity definitions (e.g., sport participation) and in populations without SUD. Prior evidence has found tobacco and cannabis use in adolescents were associated with low activity (4). Several studies have found a positive relationship between physical activity and alcohol use in adolescents (5–8). Further, Lesjak and Stanojevic-Jerkovic (9) found sedentary activities such as watching TV and using the computer for more than two hours a day were associated with heavy episodic drinking in adolescents (9). The general adult population literature suggests inverse associations between activity behaviors and problematic substance use (5), but the relationships between activity behaviors in patients with SUD remains understudied.

Little published research has described behavioral patterns or evaluated the relationships between physical activity engagement and key characteristics of SUD. SED levels may also be particularly important in people with SUD who have high depression and anxiety—common symptoms of SUD (10, 11). Drawing conclusions about the utility of SUD in a clinical setting is challenging as much of the past research has classified SUD by amount of the substance (e.g., alcoholic drinks/week) rather than using gold-standard clinical assessments of clinical SUDs. Additionally, the scant research that has attempted to evaluate behavioral patterns in SUD lacks certain analyses relevant to key characteristics related to SUD such as drug of choice (DOC) or level of care. Therefore, current research is missing information about clinical features of SUD in the adult population, which is important to understanding how physical activity engagement is related to clinically diagnosed SUD.

The present weaknesses in the literature have created a need for research that describes MVPA and SED behavior patterns, which represent two major modifiable behaviors preliminarily associated with SUD. Thus, quantification of both MVPA and SED behaviors in SUD patient populations (those initiating inpatient and outpatient SUD treatment) is a necessary step for determining the utility and parameters of future behavioral interventions. Further, additional information is needed on how activity behaviors differ by individual characteristics (e.g., sex, race, and age). Therefore, this study aims to (1) describe current self-reported MVPA and SED levels of those initiating treatment, (2) determine the potential contribution of DOC on these behaviors, and (3) determine the potential contribution of level of care and other demographic and psychological factors on these behaviors.

## 2 Methods

### 2.1 Procedures

Data was obtained from TRAC-9 Informatics (12), a software tool that was developed by a private company to allow SUD treatment facilities to monitor treatment progress, outcomes, and predict risk of treatment failure. TRAC-9 includes a standardized battery of clinical self-report assessments for patients in SUD treatment facilities across the United States that include measures associated with recovery, clinical symptoms of SUD, and mental health. De-identified data was combined from each treatment facility and exported from the TRAC-9 database. As only de-identified data was shared with the research team, the project does not meet the United States federal regulatory definition of “human subjects” research, therefore, IRB oversight was not necessary for the present study. Data was shared from August 2020 to October 2020 in patients beginning treatment (i.e., intake visit) for SUD from 27 different facilities. The software tool assesses several self-reported factors associated with successfully completing a recovery program (e.g., commitment to sobriety, anxiety symptoms, and depressive symptoms). Through a partnership with the research team, the International Physical Activity Questionnaire Short Form [IPAQ-SF; (13)] was added as a TRAC-9 standardized assessment to evaluate MVPA and SED levels. Thus, the full dataset included: demographics, DOC, level of care, MVPA and SED levels, and nine mental health questionnaires (not described or used herein). TRAC-9 Informatics had no role in the processing, analyzing, writing, or dissemination of this analysis.

### 2.2 Measures

#### 2.2.1 Demographic factors

Participants reported age, sex (the categories were labeled “Male” and “Female” will be referred to as “men” and “women”, respectively, herein), race (the category was labeled “African American” will be referred to as “Black” herein 14), ethnicity, and employment (free text responses).

#### 2.2.2 Drug of choice

Clinicians assessed preferred DOC. Response categories were alcohol, cocaine, stimulants, opioids, cannabis, methamphetamines, benzodiazepine, heroin, or other. Due to potential differences in activity level between substance type (i.e., drugs versus alcohol), alcohol was analyzed separately for Aim 2a (15, 16).

#### 2.2.3 Level of care

TRAC-9 is used by facilities across all levels of care. Thus, individual participant level of care was coded as detox, residential, Partial Hospitalization Program (PHP)/Intensive Outpatient Programs (IOP), or private practice.

#### 2.2.4 Depressive symptoms and anxiety symptoms

Depression and anxiety are common symptoms of patients with SUD. Depressive symptoms and anxiety symptoms were assessed based on established thresholds. The Center for Epidemiological Studies Depression Scale-20 [CES-D 20; α = 0.85-0.90 (17)] was used to assess depressive symptom. The questionnaire contains 20 prompts with response choices of “Rarely or none of the time (less than 1 day),” “Some or a little of the time (1-2 days),” “Occasionally or a moderate amount of time (3-4 days),” or “Most or all of the time (5-7 days).” All items were summed with total scores ranging from 0 to 60, and higher scores indicating more frequent depressive symptoms. Depressive symptoms were categorically coded into binary variables (yes/no) based on the CES-D 20 clinical cut point of mild to severe depressive symptoms (≥16 is indicative of individuals at risk for clinical depression; Aim 3). The Penn State Worry Questionnaire [α=0.88-0.95; (18)], was used to assess the trait worry, a key component of anxiety. Participants responded to 16 statements about their disposition to worry using a Likert-style scale ranging from 1 (“not at all typical of me”) to 5 (“very typical of me”). Total scores range from 16-80, indicating low (16-39), moderate (40-59) and high (60-80) worry. Similar to depressive symptoms, trait worry was categorically coded into binary variables (yes/no) based on the clinical cut point of moderate to severe trait worry (≥40 is indicative of individuals with higher trait worry; Aim 3).

#### 2.2.5 Physical activity and sedentary time

The IPAQ-SF (α = 0.80; 13) was used to assess MVPA and SED behaviors. Standard IPAQ-SF instructions were used to explain how to fill out the type of activity and the amount of time spent on activity. Patients read and responded to the following questions: “During the last 7 days, on how many days did you do [walking, moderate activity, vigorous activity] and “how much time in total per day have you spent [sitting, walking, on moderate activity, on vigorous activity]”. Prior evidence (19) has found MVPA to be associated with increased abstinence rates and decreased depressive symptoms in individuals who use drugs as well as a decrease in withdrawal and anxiety symptoms in individuals who use drugs or alcohol, therefore activity data was converted to MVPA (i.e., time spent on moderate and vigorous activity) rather than MET minutes. Walking was excluded from the analysis to minimize over reporting (20). According to the US Physical Activity Guidelines, one minute of vigorous-intensity is equivalent to 2 minutes of moderate-intensity activity. Thus, we added weekly minutes of moderate activity to twice the number of weekly minutes of vigorous activity in order to get weekly MVPA (21). For MVPA, participants were categorized based on meeting aerobic US Physical Activity Guidelines (i.e., inactive [0 min/week], insufficiently active [1-149 min/week], meets guidelines [150-299 min/week], exceeds guidelines [≥ 300 min/week] (21);. Similar to methods carried out by Ekelund et al. (22), SED was categorized into four groups based on self-reported daily SED (i.e., < 4 h/day [0-239 min/day], 4- <6 h/day [240-359 min/day], 6-8 h/day [360-480 min/day], >8 h/day [>480 min/day] (22).

### 2.3 Data cleaning process

Initially, a total of 861 moderate, 917 vigorous, and 1,672 sedentary data points were entered by participants. Physical activity and sedentary data entries (all of which had been entered using free-text) were screened to convert text responses to numeric values, where appropriate (cleaning procedures in **Supplementary File**). For example, a common error was input of non-numerical answers intended to be numerical (e.g., “60 min”, “1 hour”, “an hour”). Data entered that was unusable (e.g., answers such as “a lot” or “idk”) were excluded from the analysis. After identification of common errors, data was imported to R (23) and R (24) for data cleaning and analysis. All times for sitting, moderate, and vigorous activity were converted to minutes. As recommended by the IPAQ Research Committee (2005), standard IPAQ data cleaning rules were followed resulting in the exclusion of anyone reporting > 960 min/day of MVPA or daily SED > 1,200 min/day equivalent to 20 h/day (25). If participants did not report data for both SED and MVPA, they were excluded from the analysis resulting in the total exclusion of 367 data entries, leaving 1,293 complete data entries to be analyzed.

### 2.4 Statistical analysis

All analyses were conducted with R (23) and R Studio (24). Normality of the data was tested using a Shapiro-Wilk test with distributions of weekly self-reported MVPA (W= 0.61, *p* < 0.001) and SED (W= 0.93, *p* < 0.001) minutes departing significantly from normality. Thus, for aim 1, rates of participating in each activity behavior are described by medians and interquartile range. Percentages of participants reporting each level of behavior were calculated to describe current MVPA and SED levels by group. For aim 2, a zero-inflated Poisson regression was used to compare self-reported mean MVPA levels (26) and a t-test was used to compare self-reported mean SED levels between patients in treatment for drugs vs. alcohol. Chi-square tests were used for aims 2 and 3 to determine if proportions of participants self-reporting each activity behavior level were different based on individual DOC (Aim 2), level of care, psychological health, and demographic factors (Aim 3). Differences in activity based on DOC were first examined by DOC group (i.e., alcohol vs. all drugs) and then by specific DOC (Aim 2). One assumption of a Chi-square test is every level of a group has an adequate sample size with a cell expected value at 5 or more and no cell should have an expected value of less than one (27). Individual (n=2; *Level of Care*), the assignment of a sex other than male or female (n=2; *Sex*), and the age groups of 65-74 (n= 22) and 75+ (n= 3; *Age*) violated the cell expected value assumption and were subsequently dropped from this analysis. Further, to satisfy the cell expected value assumption, people who identified as Asian (n=6), Native American (n=16), and Native Hawaiian/Pacific Islander (n=5) were added into “other” of the *Race* group. Post-hoc chi-square tests were conducted to determine significant pairwise differences (Aim 2 and Aim 3; α = 0.05). The analyses conducted within this manuscript have not been pre-registered and the results should be considered exploratory.

## 3 Results

A majority of participants with usable data (n=1,293) were White (87%), men (66%), between the ages of 25-34 (27%). Most of the participants in this analysis were receiving residential care (70%), entering treatment for alcohol use disorder (52%), and many reported depressive symptoms (80%) and anxiety symptoms (81%). Chi-square analyses revealed no statistically significant difference between people who were included vs. excluded in the analysis based on demographics (*p* > 0.05). Full study sociodemographic characteristics are classified by MVPA and SED behaviors in **Table 1**.

**Table 1:** Sociodemographic Characteristics of Participants by Activity Behavior.

	N	MVPA n (%)				SED n (%)			
		Inactive	Insufficiently Active	Meets Guidelines	Exceeds Guidelines	<4 h/day	4-<6 h/day	6-8 h/day	>8 h/day
Level of Care									
Detox	310	151 (48%)^a^	36 (12%)	30 (10%)	93 (30%) ^a^	112 (36%)	56 (18%)	55 (18%)	87 (28%)
IOP/PHP	72	21 (29%) ^b^	7 (10%)	2 (3%)	42 (58%) ^b^	32 (44%)	17 (24%)	12 (17%)	11 (15%)
Residential	909	334 (37%) ^b^	110 (12%)	89 (10%)	376 (41%) ^a,b^	301 (33%)	211 (23%)	170 (19%)	227 (25%)
Individual*	2	0 (0%)	0 (0%)	0 (0%)	2 (100%)	2 (100%)	0 (0%)	0 (0%)	0 (0%)
Age									
16-24	211	70 (33%) ^b^	29 (14%)	18 (8%)	94 (45%)	79 (37%)	44 (21%)	44 (21%)	44 (21%)
25-34	345	125 (36%) ^b^	48 (14%)	32 (9%)	140 (41%)	114 (33%)	69 (20%)	61 (18%)	101 (29%)
35-44	341	126 (37%) ^b^	34 (10%)	40 (12%)	141 (41%)	126 (37%)	89 (26%)	50 (15%)	76 (22%)
45-54	236	103 (44%) ^b^	24 (10%)	18 (8%)	91 (38%)	74 (31%)	48 (20%)	51 (22%)	63 (27%)
55-64	135	69 (51%) ^a^	15 (11%)	13 (10%)	38 (28%)	49 (36%)	25 (19%)	27 (20%)	34 (25%)
65-74*	22	11 (50%)	3 (14%)	0 (0%)	8 (36%)	4 (18%)	9 (41%)	4 (18%)	5 (23%)
75+*	3	2 (67%)	0 (0%)	0 (0%)	1 (33%)	1 (33%)	0 (0%)	0 (0%)	2 (67%)
Sex									
Male	854	309 (36%) ^a^	93 (11%)	73 (9%)	379 (44%) ^a^	325 (38%) ^a^	191 (22%)	152 (18%)	186 (22%) ^a^
Female	437	196 (45%) ^b^	60 (14%)	48 (11%)	133 (30%) ^b^	122 (28%) ^b^	93 (21%)	85 (20%)	137 (31%) ^b^
Other*	2	1 (50%)	0 (0%)	0 (0%)	1 (50%)	0 (0%)	0 (0%)	0 (0%)	2 (100%)
Race									
White	1,130	447 (40%)	139 (12%)	106 (9%)	438 (39%)	372 (33%) ^a^	256 (23%)	205 (18%)	297 (26%)
Black	63	24 (38%)	7 (11%)	8 (13%)	24 (38%)	34 (54%) ^b^	5 (8%)	13 (21%)	11 (17%)
Other	100	35 (35%)	7 (7%)	7 (7%)	51 (51%)	41 (41%) ^a,b^	23 (23%)	19 (19%)	17 (17%)
Ethnicity									
Hispanic/Latino	93	41 (44%)	9 (10%)	3 (3%)	40 (43%)	33 (36%)	19 (20%)	17 (18%)	24 (26%)
Not Hispanic/Latino	1200	465 (39%)	144 (12%)	118 (10%)	473 (39%)	414 (35%)	265 (22%)	220 (18%)	301 (25%)
Employment									
Employed	594	212 (36%)	72 (12%)	54 (9%)	256 (43%)	204 (34%)	139 (23%)	104 (18%)	147 (25%)
Unemployed	699	294 (42%)	81 (11%)	67 (10%)	257 (37%)	243 (35%)	145 (21%)	133 (19%)	178 (25%)
Depression									
Yes	1,039	429 (41%) ^a^	127 (12%)	99 (10%)	384 (37%) ^a^	345 (33%)	215 (21%)	194 (19%)	285 (27%) ^a^
No	254	77 (30%) ^b^	26 (10%)	22 (9%)	129 (51%) ^b^	102 (40%)	69 (27%)	43 (17%)	40 (16%) ^b^
Anxiety									
Yes	1,049	419 (40%)	117 (11%)	101 (10%)	412 (39%)	369 (35%)	221 (21%)	187 (18%)	272 (26%)
No	244	87 (36%)	36 (15%)	20 (8%)	101 (41%)	78 (32%)	63 (26%)	50 (20%)	53 (22%)

MVPA, moderate-vigorous physical activity; SED, sedentary; IOP, intensive outpatient programs; PHP, partial hospitalization program.

Cells that are marked that share a superscript letter are not significantly different from each other according to the chi-square analysis from Aim 3.

^a,b^ Comparisons are being made within the group within the column (e.g., inactive, detox is significantly different from inactive, IOP/PHP for MVPA).

*Due to a small cell count, individual facility (level of care), 65-75+ (age), and other (sex) were dropped from the Aim 3 analysis and racial groups other than.

White and Black were combined into an “other” category.

### 3.1 Aim 1: current MVPA and SED levels

**Figure 1:**
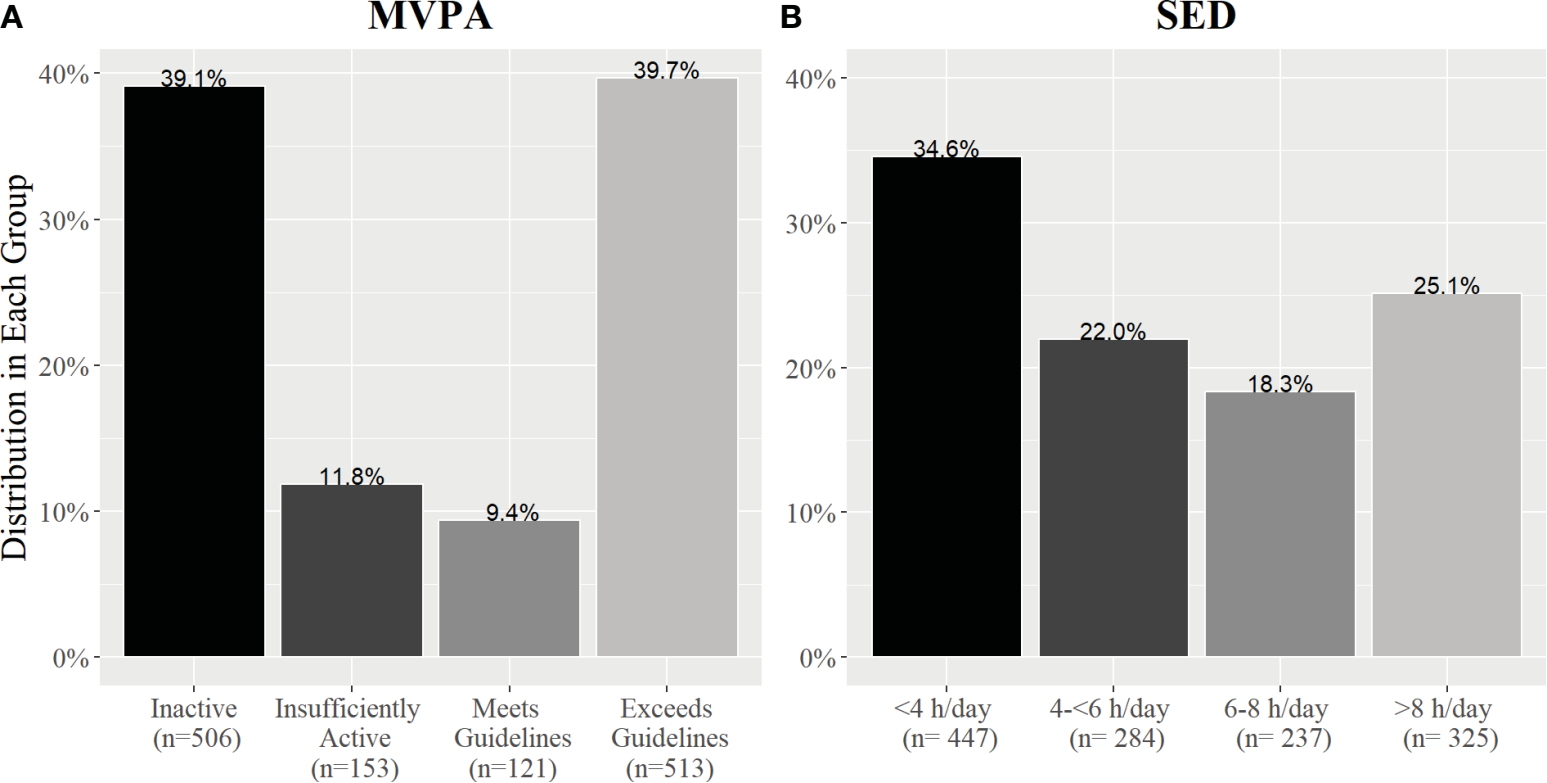
The percentage of adults with SUD in each activity group based on self-reported **(A)** weekly MVPA and **(B)** daily SED. Panel A shows the percentage of people in each MVPA group based on meeting the US Physical Activity Aerobic Guidelines and Panel B shows the percentage of people in each SED group based on the number of hours per day they self-reported sitting.

Median self-reported MVPA time was 120 min/week (IQR: 0-630 min/week) and median SED level was 360 min/day (IQR: 180-540 min/day). Participants reporting each level of behavior of MVPA and SED are presented in **Figure 1**.

### 3.2 Aim 2a: alcohol vs. drugs and activity behaviors

**Figure 2:**
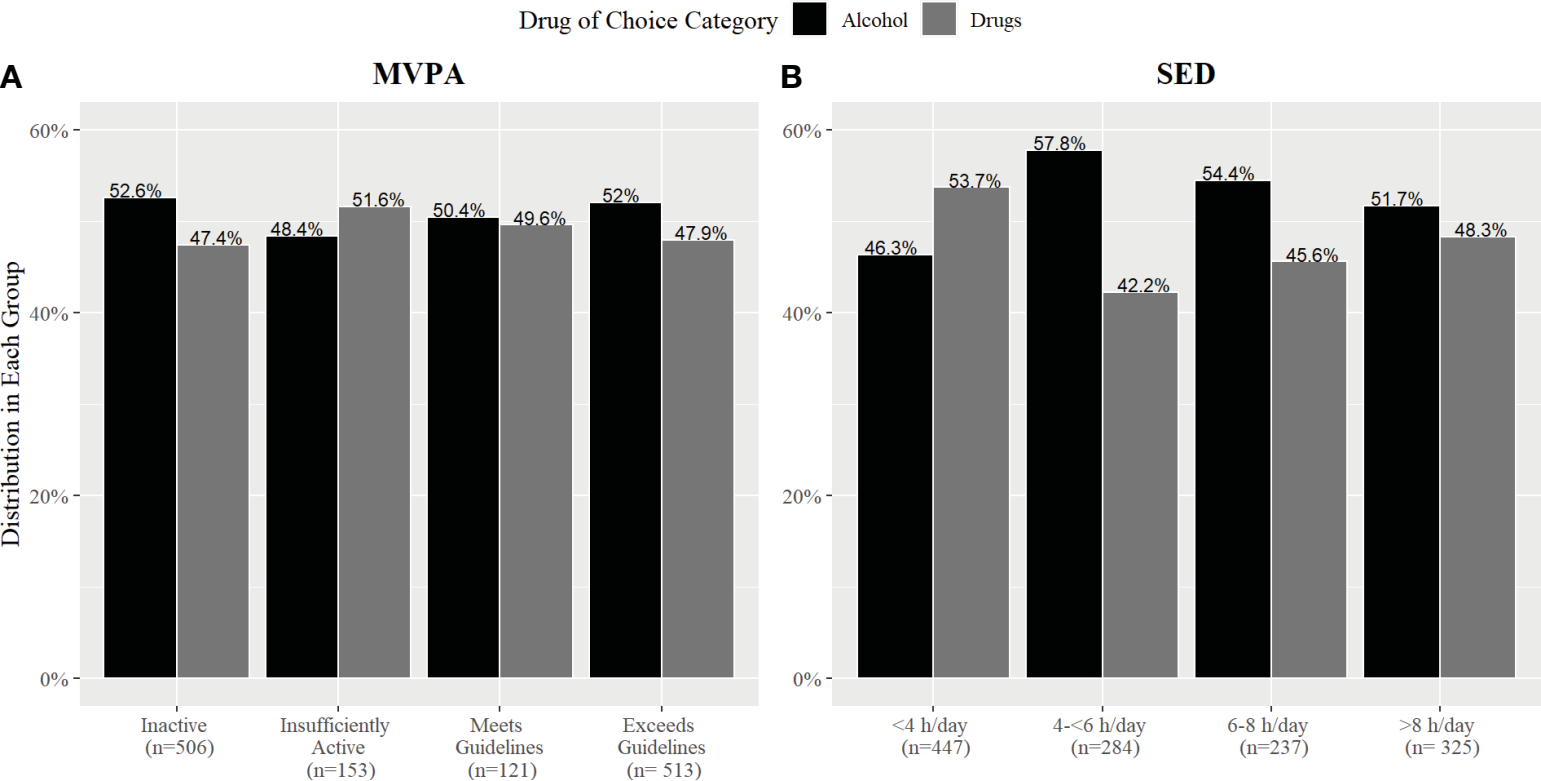
The percentage of adults with SUD in each activity group based on self-reported **(A)** MVPA and **(B)** SED by drug of choice (DOC; alcohol vs. drugs).

There was no significant difference in MVPA time for patients admitted for drug use (mean*=* 535.58 min/week ± 935.24 standard deviation) compared to alcohol use (486.18 min/week ± 852.15; *β*= -0.05, p= 0.61). There was no significant difference in average SED time for patients admitted for drug use (395.44 min/day ± 290.64) compared to alcohol use (*M=* 396.52 min/day ± 261.01; *t*(1253.4)= 0.06, p= 0.94). **Figure 2** shows the distribution of people taking drugs compared to alcohol in each activity group. There was no significant association between the percentage of people in each MVPA group and DOC group (alcohol vs. drugs: χ^2^(3, 1293)= 0.94, p=0.81, V= 0.03). There was a statistically significant relationship between DOC group based on SED group with a small to medium effect size (χ^2^(3, 1293)= 10.06, p=0.01, V= 0.08).

### 3.3 Aim 2b: individual drugs and activity behaviors

**Figure 3:**
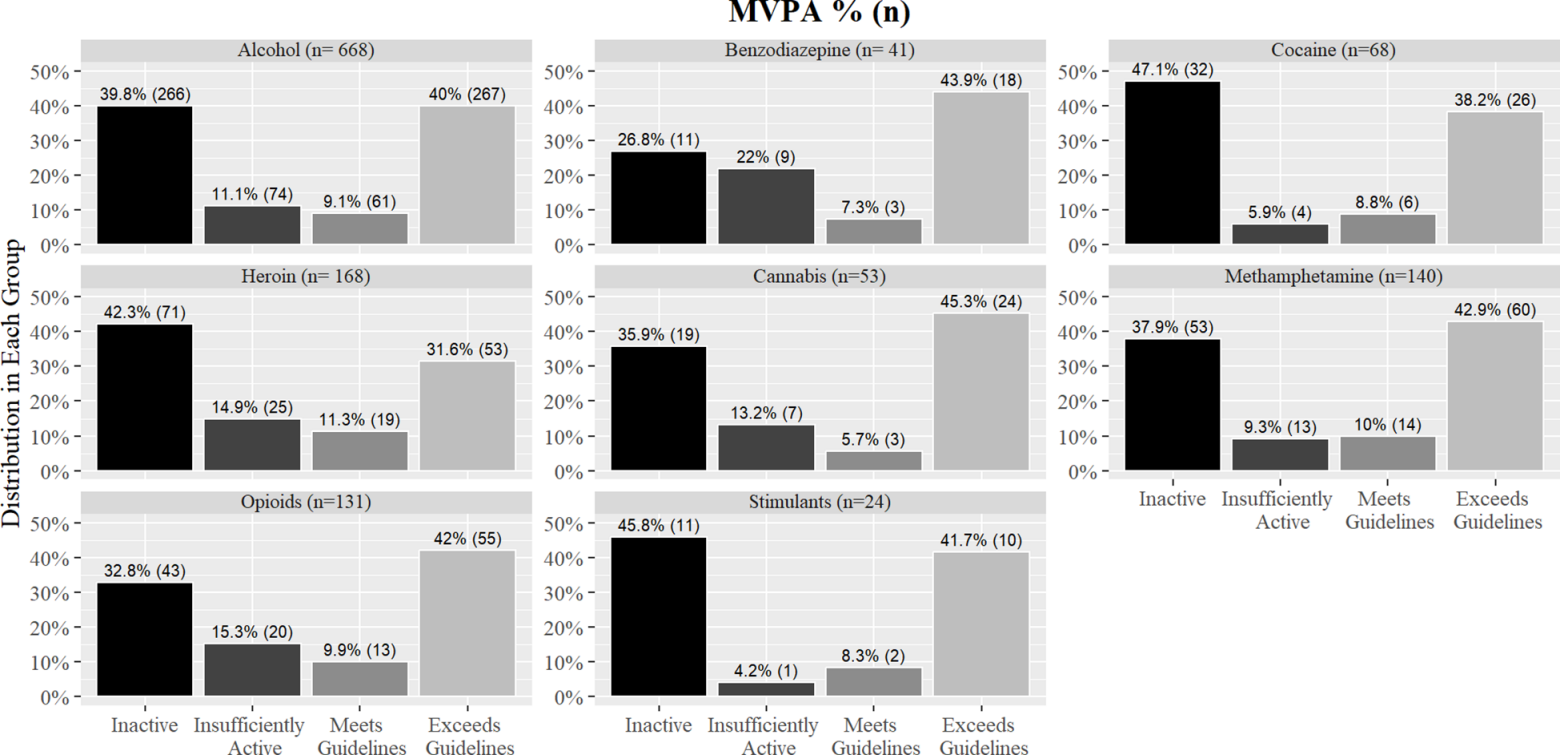
Percentages of MVPA groups by individual drug of choice.

**Figure 4:**
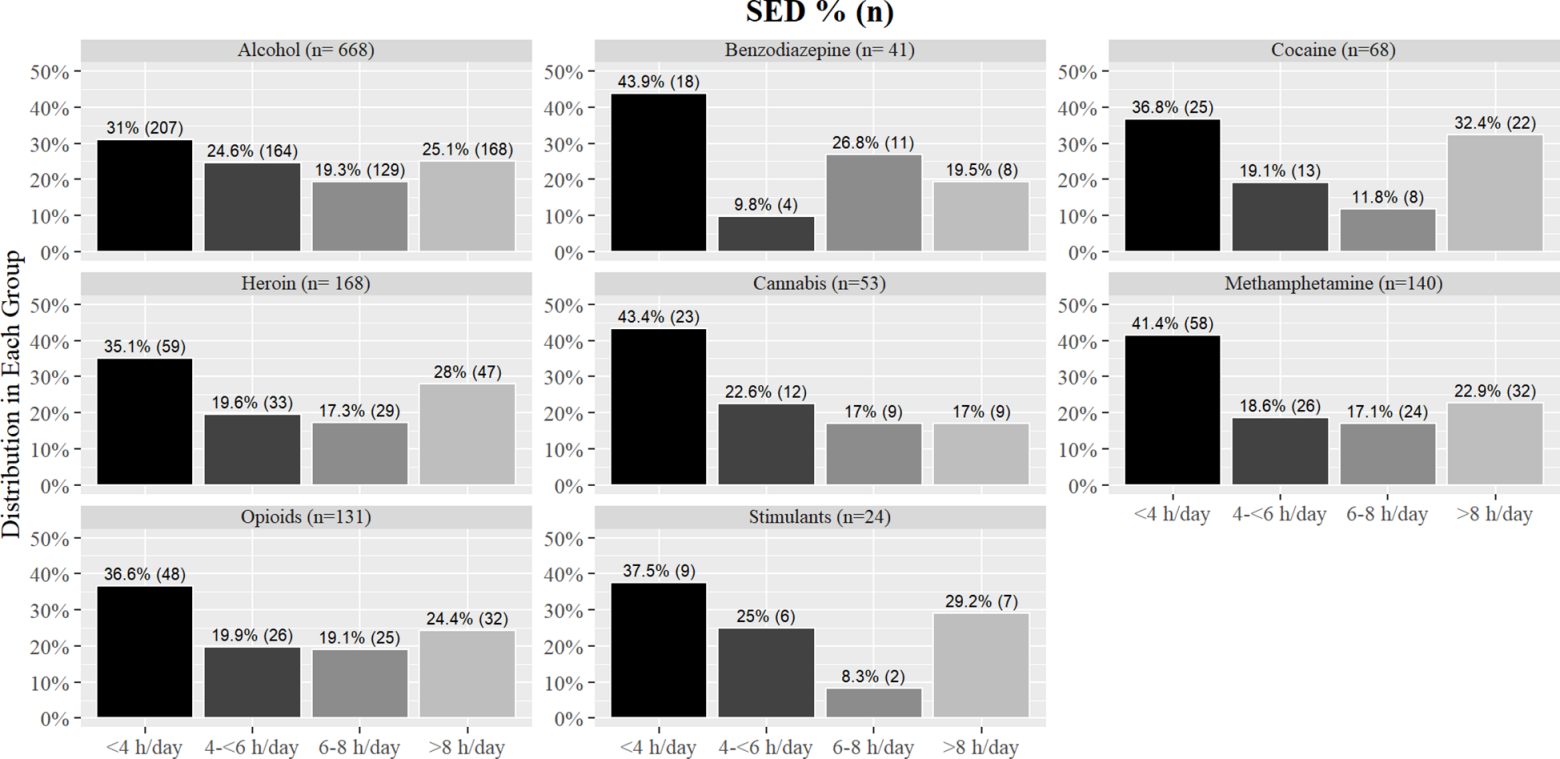
Percentages of SED groups by individual drug of choice.

There was no significant difference between the percentage of people in each group for either MVPA (χ^2^ (21, 1293) = 21.40, *p* = 0.43, V= 0.07; **Figure 3**) or SED (χ^2^ (21, 1293) = 23.05, *p* = 0.34, V= 0.07; **Figure 4**) when participants were separated based on each DOC.

### 3.4 Aim 3: demographic and activity behaviors

Overall model results including significant pairwise differences are presented in **Table 1**. There was a significant difference in the percentage of people in each MVPA group based on level of care (χ^2^ (6, 1291) = 28.59, p < 0.001, V= 0.09) and sex (χ^2^ (3, 1291) = 23.52, p < 0.001, V= 0.13). Significant differences for MVPA levels emerged for age (χ^2^ (12, 1268) = 21.84, p = 0.03, V= 0.05). There was a significant difference in the percentage of people in each MVPA group based on depressive symptoms (χ^2^ (3, 1293) = 16.96, p < 0.001, V= 0.10). No significant differences emerged for race (χ^2^ (6, 1293) = 7.57, p = 0.27, V= 0.02), ethnicity (χ^2^ (3, 1293) = 5.32, p= 0.14, V= 0.04), employment (χ^2^ (3, 1293) = 6.73, p = 0.08, V= 0.05), and anxiety symptoms (χ^2^ (3, 1293) = 3.75, p = 0.28, V= 0.02).

There was a significant difference in the percentage of people in each SED group based on sex (χ^2^ (3, 1291) = 19.74, p < 0.001, V= 0.11), race (χ^2^ (6, 1293) = 19.81, p = 0.002, V= 0.07), and depressive symptoms (χ^2^ (3, 1293) = 18.16, p < 0.001, V= 0.11). No significant results of SED levels emerged for level of care (χ^2^ (6, 1291) = 9.76, p = 0.13, V= 0.04), age (χ^2^ (12, 1268) = 16.59, p = 0.16, V= 0.03), ethnicity (χ^2^ (3, 1293) = 0.14, p = 0.98, V= 0.01), employment (χ^2^ (3, 1293) = 1.51, p = 0.67, V= 0.03), and anxiety symptoms (χ^2^ (3, 1293) = 4.78, p = 0.18, V= 0.04).

## 4 Discussion

This study examined rates of physical activity and sedentary behavior among individuals entering treatment for SUD and their distribution across demographic and clinical factors. The key findings in our analysis include: (1) approximately 51% of patients entering treatment self-reported not meeting the aerobic activity guidelines, but most (57%) reported sitting less than 6 hours per day; (2) there was no significant difference between average MVPA and SED time for patients admitted for drugs vs. alcohol and or among different drug types; and (3) MVPA groups differed by level of care, with detox facilities having a higher percentage of physically inactive patients compared to residential facilities and IOP/PHP. Women were more likely to report physical inactivity compared to men, while Black patients were more likely to report low sitting time (<4 h/day) compared to White patients or other races. Finally, those reporting depressive symptoms were more likely to be inactive and report high levels of sitting time (> 8 h/day) compared to those who did not report depressive symptoms. Overall, the present study’s results provide strong descriptive epidemiological data regarding current activity behaviors from a large sample entering treatment for SUD.

Median self-reported MVPA was 120 min/week (n=1,293)—insufficient to meet the recommended 150 min/week. About half of the patients (51%) reported not meeting the guidelines, while the other half (49%) met or exceeded them. Our data exhibited a U-shaped association (i.e., largest numbers self-reporting amounts in the smallest and largest categories of MVPA and SED), similar to a previous large-scale observational study analyzing the association between physical and mental health, where many participants reported low or high activity levels with relatively few participants reporting amounts of activity in between (28). Additionally, as of 2018, 53.3% of adults over the age of 18 reported meeting the US Physical Activity Guidelines, aligning closely with our distribution (29). Moreover, our analysis revealed a median SED of 6 h/day. This finding a comparable to the results of a serial, cross-sectional analysis of the National Health and Nutrition Examination Survey 2001-2016 where Yang et al. (30) found the average self-reported SED time for US adults was 5.2-5.6 h/day (30). Taken together, these findings show activity levels of patients entering treatment for SUD are similar to the general population.

While there were no significant differences in mean MVPA and SED between patients admitted for drugs compared to alcohol, it is notable patients in treatment for alcohol reported higher sitting times across SED groups compared to patients in treatment for drug use. Previous research has shown people who consume hazardous amounts of alcohol are more likely to have high sedentary time in their leisure time than those who drink a low amount of alcohol (31). However, the relationship between drug use and activity behaviors is understudied. In the current analysis, we combined all drugs together and compared them with alcohol use as there is evidence that suggests alcohol is associated with physical activity differently than drugs (5). Further, high physical activity is associated with high alcohol intake in most general adult populations, though less is known of this association among clinical alcohol use disorder (32). Therefore, this associative difference might be applicable to sedentary behavior as well. However, when the analysis was conducted across the individual drugs, there was insufficient evidence to identify relationships between the individual substances and activity behaviors. In our study, a majority of patients were in treatment for alcohol use, therefore is it plausible the low frequency of patients in the different drug categories was not enough to find an association between individual drugs and activity behaviors.

In the current analyses we analyzed how several different demographic factors related to activity behaviors. Within the level of care, individuals entering detox facilities were more likely to self-report inactivity compared to those entering residential facilities, and individuals entering IOP/PHP facilities were more likely to self-report exceeding the guidelines compared to those entering detox facilities. According to the American Society of Addiction Medicine, the level of care provided to the patient depends on several criteria (i.e., severity of addiction, behavioral control, readiness for change, etc; 33). Further, the severity of the patients’ medical history may influence the required level of care, potentially limiting their ability to engage in exercise (33). For example, patients that are entering detox facilities may have more serious medical limitations that prevent them from exercise compared to patients who are entering outpatient facilities. Therefore, the activity behavioral profiles of each patient that enters the different treatment facilities could be associated with the nature and/or severity of the addiction and the patients’ medical history.

In addition to level of care, sex was associated with self-reported activity level. In our findings, women were more likely to report inactivity and high sitting time (>8 h/day) compared to men, which is a pattern found in the general adult population (34). Prior research has found that SUD affects men and women differently. McHugh et al.’s (35) systematic review found certain biological factors (e.g., hormones) can influence the acute effects of substance use and the response to treatments. Further, they found SUDs are more severe in women than in men as women appear to be more likely to report using substances to help alleviate anxiety symptoms (35). However, given that the present study is a descriptive analysis, it is important to acknowledge the potential for multiple factors contributing to our results, thus potentially raising a confounding concern. For example, existing literature has highlighted that women with addictive disorders tend to exhibit higher levels of depressive symptoms (36). Consequently, a logical next step would involve conducting analyses that effectively control for confounding variables. Additionally, Black patients were more likely to report low sitting time (<4 h/day) compared to White patients or patients from other racial groups. This result is inconsistent with the findings of Yang et al. (30) who found Black individuals in the general adult population were at a higher risk of prolonged sitting time than White individuals (30). Conversely, while Cohen et al. (37) found Black individuals are more likely to spend a longer amount of time engaged in certain sedentary behaviors (i.e., watching television) compared to White individuals, there was a minimal difference found for overall daily sedentary behaviors in these participants entering SUD treatment (37). Overall, our findings suggest that both the DOC and demographic backgrounds may contribute to physical activity and sedentary behaviors in SUD.

Finally, we found individuals who reported depressive symptoms were more likely to be inactive, less likely to exceed guidelines, and more likely to report high SED levels (>8 h/day) compared to those who reported no depressive symptoms. Prior evidence has confirmed adults with depressive symptoms tend to engage in low levels of physical activity and report high levels of sedentary behavior (38), therefore the results of our study were in line with previous research. Further, as rates of depressive symptoms are typically high among people with SUD (39), it is unsurprising that 80% of our respondents reported sufficient frequency of symptoms to be indicative of clinical depression.

### 4.1 Limitations

This analysis has several limitations to consider. Firstly, is should be noted that our study is based on a cross-sectional analysis of patients entering treatment. Additionally, our research predominately focuses on aerobic activity, which only accounts for half of the US Physical Activity Guidelines, as the guidelines also recommend a minimum of 2 days/week of muscle-strengthening activities. Both SED and MVPA levels were self-reported, and people tend to over report MVPA and underreport SED. We attempted to minimize inaccurate self-reporting by following established protocol in data analysis (25). These results represent levels of behavior upon arrival for treatment, therefore we cannot make conclusions about physical activity behaviors within treatment for SUD. It is likely that because activity assessments were taken at baseline, patients may be conflating some of their initial activity time at the treatment facility with their activity in the few days prior to treatment, given that the IPAQ is a 7-day recall assessment. However, despite these limitations, describing baseline MVPA and SED levels in a large sample of treatment-initiating patients provides the foundation for determining the potential room to change MVPA/SED in SUD treatment in the future.

## 5 Conclusions

Our findings show MVPA and SED patterns of SUD patients upon treatment initiation hold significant room to increase MVPA and decrease SED levels (similar to the general adult population where both behaviors are key lifestyle behavior targets). As the association of activity behaviors and individual drug of choice was unclear, more research is needed to determine how individual substances may influence activity behaviors. Due to differences in behavioral patterns based on DOC, level of care, psychological factors, and demographics, treatment facilities might be able to target behavioral programs to specific subgroups with especially low likelihoods of current activity participation or high SED (e.g., women, patients in detox facilities, patients with depressive symptoms). Overall, activity behavior patterns in patients entering treatment for SUD may offer insight into assessing lifestyle behavior needs and developing personalized treatment strategies.

## Data availability statement

The data analyzed in this study is subject to the following licenses/restrictions: This data was from a partnership with a company. Requests to access these datasets should be directed to Trac9 contact@trac9.com.

## Ethics statement

Ethical approval was not required for the study involving humans in accordance with the local legislation and institutional requirements. Written informed consent to participate in this study was not required from the participants or the participants’ legal guardians/next of kin in accordance with the national legislation and the institutional requirements. Written informed consent was obtained from the individual(s) for the publication of any potentially identifiable images or data included in this article.

## Author contributions

SC: Conceptualization, Data curation, Formal analysis, Investigation, Methodology, Software, Visualization, Writing – original draft, Writing – review & editing. JL: Conceptualization, Formal analysis, Investigation, Software, Supervision, Visualization, Writing – original draft, Writing – review & editing. AB: Conceptualization, Data curation, Formal analysis, Investigation, Supervision, Validation, Visualization, Writing – original draft, Writing – review & editing. JM: Conceptualization, Data curation, Formal analysis, Investigation, Project administration, Resources, Software, Supervision, Validation, Visualization, Writing – original draft, Writing – review & editing.
