# Supplementary material for: Epidemiology of physical activity and sedentary behavior levels among patients entering treatment for substance use disorder in the United States: a descriptive study
Source: Front Psychiatry. 2024 Apr 24;15:1348047. doi: 10.3389/fpsyt.2024.1348047 (PMC11076814; doi:10.3389/fpsyt.2024.1348047)
Supplement: Supplementary file 1 [file Presentation_1.pdf]

Supplementary Material

Epidemiology of physical activity and sedentary behavior levels among patients entering treatment for substance use disorder

Sydney L. Churchill, MA[1], Jeni E. Lansing, PhD[1] , Angelique G. Brellenthin, PhD[1], Jacob D. Meyer, PhD[1*]

[1]Department of Kinesiology, Iowa State University, Ames, IA, United States

*** Correspondence:**
Jacob D. Meyer
jdmeyer3@iastate.edu

1 Data Cleaning Process

The International Physical Activity Questionnaire-Short Form (IPAQ) was added to the TRAC-9 standardized assessment to evaluate activity and sedentary behavior by anyone completing the TRAC-9 tool, typically patients in SUD treatment. In the present dataset, this assessment was completed via open-ended response questions across 12 time points of treatment, therefore data cleaning was necessary in order to convert the open-ended entries to numeric values for data-processing. This supplementary file outlines data cleaning steps taken in R [1], R studio [2], and Microsoft Excel. The following document describes the processing of the baseline data time point, with all steps repeated for all 11 subsequent time points.

In order to identify usable data for the sitting variable, an initial summary was coded to compare counts for numeric data, blank data, and data that was in a string text format. Non-numeric data was then examined to determine if it could be converted to usable data. Responses were identified that included string text and converted into numeric responses. Due to the open ended nature of the response type, some numeric entries would also contain a text description. For example, "4 hours" instead of "4". Entries entered as hours were converted to minutes by multiplying them by 60. Several variations of the word "hours" (hours, hour, hrs, hr) were used with each converted to minutes. Similarly to entries containing the word "hours" in responses, some entries also contained the word "minutes" after the numeric response (e.g., 45 minutes instead of 45). All patterns of the word minutes (minutes, minute, mins, min) were removed in an identical fashion to the hours processing. After common errors were fixed, "unique" data that did not fit the above criteria was evaluated. For example, some entries contained misspellings of the words hour or minute, used the word "roughly", "+" or "a day", or numeric entries were spelled out (i.e., responding "two" instead of 2").

It was assumed that entries of the numbers 1-5 were intended to be hours and that 6-9 were minutes. Moreover, per IPAQ Truncation Rules [3], anything less than 10 minutes must be reassigned to 0. Therefore an assumptions column was made for future conversion steps and everything in the assumptions column was initially dummy coded to 0. Daily minutes were then capped to a 960 minutes/day maximum per IPAQ Truncation Rules and the number of days in the week were capped

at a 7 day maximum. To calculate MVPA, total weekly minutes for vigorous activity was multiplied by 2 and the added to total weekly minutes of moderate activity. Vigorous is doubled to make it equivalent to moderate time per the Physical Activity Guidelines. The outline cleaning process was repeated for all 12 time points.

After this, the data was categorically coded. A code was created to determine the interquartile range for SED to determine the correct categorical distribution. Next, SED was categorically coded for all 12 time points. This was based on the interquartile range selected from Ekelund et al. (2016). The categories included: 0-4 hours (0-240 minutes), 4-6 hours (241-360 minutes), 6-8 hours (361-480 minutes), and 8+ hours (≥ 481 minutes)[4]. Next, MVPA data was coded based on the U.S. Aerobic Physical Activity Guidelines as follows: inactive (0 minutes), insufficiently active (1-149 minutes), meets guidelines (150-299 minutes), and exceeds guidelines (≥ 300 minutes)[5]. This was repeated for all 12 time points.

To ensure that everything was coded correctly, the cleaned dataset was exported to excel and reviewed for again. Each activity column (sitting, walking, moderate, and vigorous) for all 12 time points was cross-referenced with the original dataset. A column was made to indicate if the cells were manually cleaned. If the cells were manually updated, a 1 was assigned to them and if they were not, they were assigned a 0. The manually cleaning process involved: (1) updating cells that R had missed. For example, if an entry contained any variation of the words hour/minutes in the cells but it was spelled wrong, then this was updated as the correct numeric value, (2) fixing times that did not match the original dataset and (3) taking the midpoint of answers that provided ranges (eg., 20-30 minutes was changed to 25 minutes). This was done for all 12 time points to ensure accuracy of the data and then was save and imported back in the R script to get update MVPA and SED values.

References

1. R Core Team. A language and environment for statistical computing. Published online 2018. http//www.R-project.org/

2. R Studio Team. R Studio: Integrated development for R. Published online 2015. http://www.rstudio.com/

3. IPAQ Research Committee. Guidelines for data processing and analysis of the International Physical Activity Questionnaire (IPAQ)-short and long forms. Published online 2005. http://www.ipaq.ki.se/scoring.pdf.

4. Ekelund U, Steene-Johannessen J, Brown WJ, et al. Does physical activity attenuate, or even eliminate, the detrimental association of sitting time with mortality? A harmonised meta-analysis of data from more than 1 million men and women. *Lancet Lond Engl*. 2016;388(10051):1302-1310. doi:10.1016/S0140-6736(16)30370-1

5. US Department of Health and Human Services. Physical Activity Guidelines for Americans, 2nd edition. Published online 2018:118.
